# East Texas Medico-Chirurgical Society

**Published:** 1901-11

**Authors:** 


### East Texas Medico-Chirurgical Society.

The East Texas Medico-Chirurgical Society will meet Thursday and Friday, November 28th and 29th, 1901, at Palestine, Texas.

### OFFICERS ELECT.

Dr. W. C. Lipscomb, President, Crockett, Texas.
Dr. E. E. Guinn, Secretary, Rusk, Texas.

### PROGRAM.

#### FIRST DAY, THURSDAY, NOVEMBER 28TH, 2 P. M.

1. Call to order by Dr. E. B. Parsons, of Committee on Arrangements.
2. Invocation. Rev. R. H. Crozier.
3. Welcome Address. Hon. B. S. Gardner.
4. Response. Dr. W. C. Lipscomb.
5. Roll Call. Reading Minutes of Last Meeting.
6. Installation of Officers.
7. Application for Membership.
8. Report of Committees.
9. Payment of Dues.

Adjournment until 8 p. m.

#### FIRST DAY, EVENING SESSION.

1. Call to Order by the President.
2. Reading Papers—Section on General Medicine—Dr. J. H. Joyce, Chairman, Buffalo.
3. Epidemic Influenza. By Dr. J. H. Paxton, Elkhart.
4. Typhoid Fever and Its Treatment. By S. N. Aston, Jewett.

5. Alcohol and its use. By Dr. E. E. Guinn, Rusk.
6. Adjourned to Banquet.

SECOND DAY, MORNING SESSION, 9 A. M.

1. Call to Order by the President.
2. Bills, Communications, etc., Section on General Surgery, by Dr. E. W. Link, Chairman, Palestine.
3. Naevi, Report of Case and Treatment. By Dr. R. M. Dunn, Palestine.
4. Ligation of Femoral Artery After Great Loss of Blood—Intravenous Transfusion Saline Solution. By Dr. E. E. Guinn, Rusk.
5. Section on Obstetrics and Gynecology, Dr. J. T. Bell, Chairman, Tyler.
6. Leucorrhea and its Treatment. By Dr. Robt. Y. Lacy, Pittsburg.
7. Management of Normal Labor, and After Treatment. By Dr. B. F. Chambers, Palestine.
8. Should Infants be Fed First Three Days After Birth. By Dr. J. L. Hall, Crockett.
9. Symptoms and Treatment of Gonorrhea in the Female. By Dr. E. B. Parsons, Palestine.

Adjourned until 2 p. m.

AFTERNOON SESSION, SECOND DAY.

1. Call to Order by the President.
2. Section on Diseases of Children and Therapeutics, Dr. L. Merriwether, Grapeland.
3. Cholera Infantum. By Dr. F. B. Moore, Brushy Creek.
4. Child's Second Summer. By Dr. Sam R. Burroughs, Buffalo.
5. Unfinished Business.

Adjournment.

COMMITTEE OF ARRANGEMENTS.

Dr. A. L. Hathcock, Chairman, Palestine, Texas; Dr. E. W. Link, Dr. E. B. Parsons.

